# Comparison of correctly and incorrectly classified patients for in-hospital mortality prediction in the intensive care unit

**DOI:** 10.1186/s12874-023-01921-9

**Published:** 2023-04-24

**Authors:** Eline Stenwig, Giampiero Salvi, Pierluigi Salvo Rossi, Nils Kristian Skjærvold

**Affiliations:** 1 Department of Circulation and Medical Imaging, The Norwegian University of Science and Technology, Trondheim, Norway; 2 Department of Electronic Systems, The Norwegian University of Science and Technology, Trondheim, Norway; 3 KTH, Royal Institute of Technology, EECS, Stockholm, Sweden; 4 Clinic of Anaesthesia and Intensive Care Medicine, St. Olav’s University Hospital, Trondheim, Norway

**Keywords:** Machine learning, Explainability, Mortality prediction, eICU, SHAP values

## Abstract

**Background:**

The use of machine learning is becoming increasingly popular in many disciplines, but there is still an implementation gap of machine learning models in clinical settings. Lack of trust in models is one of the issues that need to be addressed in an effort to close this gap. No models are perfect, and it is crucial to know in which use cases we can trust a model and for which cases it is less reliable.

**Methods:**

Four different algorithms are trained on the eICU Collaborative Research Database using similar features as the APACHE IV severity-of-disease scoring system to predict hospital mortality in the ICU. The training and testing procedure is repeated 100 times on the same dataset to investigate whether predictions for single patients change with small changes in the models. Features are then analysed separately to investigate potential differences between patients consistently classified correctly and incorrectly.

**Results:**

A total of 34 056 patients (58.4%) are classified as true negative, 6 527 patients (11.3%) as false positive, 3 984 patients (6.8%) as true positive, and 546 patients (0.9%) as false negatives. The remaining 13 108 patients (22.5%) are inconsistently classified across models and rounds. Histograms and distributions of feature values are compared visually to investigate differences between groups.

**Conclusions:**

It is impossible to distinguish the groups using single features alone. Considering a combination of features, the difference between the groups is clearer. Incorrectly classified patients have features more similar to patients with the same prediction rather than the same outcome.

**Supplementary Information:**

The online version contains supplementary material available at 10.1186/s12874-023-01921-9.

## Background

Patient mortality predictions by statistical and machine learning (ML) modelling have been a studied topic for many years, with potential future clinical uses being patient triage, resource allocation, determining levels of care, and as a basis for End-of-Life decisions and conversations with the patient and their relatives [1]. Traditionally, severity of illness scores based on ‘classical’ statistical modelling have been used, e.g., the Acute Physiology and Chronic Health Evaluation (APACHE) I-IV, the Simplified Acute Physiology Score (SAPS) I-III, and the Mortality Probability Model (MPM) [2]. With the increasing amount of data and computational power, ML modelling is becoming increasingly popular in patient mortality predictions, and is named one of the focus areas for big data within health care [3].

Despite promising results in research papers, ML mortality prediction models have so far failed to be implemented in clinical practice [4]. One important factor concerning the use of ML methods in health care settings is the concept of ‘trust’ [5, 6]. Two terms closely related to this concept are explainablility and interpretability. These terms are often used interchangeably, and their definitions differ between papers. However, an important part of both is understanding why models make a certain prediction. We want to assure that a model predicts the correct output for the right reasons, and not because of inappropriate confounding variables or biases. In addition, understanding why and how models make their decisions aid knowledge derivation from patterns in data that are not readily available for humans [7, 8]. Knowledge regarding which samples a model is more or less reliable for is imperative for making informed decisions. There could be several reasons why an ML prediction model does not perform as expected; related to the data, the model itself, as well as the representation and interpretation of the results. Uncertainty in ML models stems from many sources, such as data inaccuracy, incomplete domain coverage and inaccurate models. The total uncertainty of an ML model comprises the aleatoric and epistemic uncertainties. Aleatoric uncertainty is due to the natural stochasticity of the observations and is an inherent irreducible uncertainty within the dataset while the epistemic uncertainty is due to a lack of data and knowledge that, in theory, can be reduced by adding more samples or features, or changing the type of model or model parameters [9]. At some point, it is impossible to further improve the results regardless of the model since the data themselves do not contain sufficient information.

Knowing the limitations of a model is crucial. No model is perfect and this needs to be accounted for when using them. Patients are a very heterogeneous group, and the individual differences are large. With this study we want to investigate whether individual features could be used to explain why some patients are classified incorrectly, regardless of which model is used. By developing ML models predicting hospital mortality in intensive care unit (ICU) admissions and comparing the results, this study explores differences and similarities between patients that are consistently correctly or incorrectly classified. Combining the results from three different ML algorithms and repeating the training and testing multiple times reduces some of the epistemic uncertainty related to the specific algorithms and inaccuracies and provides more generalisable results compared to the analysis of a single model. A comparative analysis of the feature importances and values is then conducted to see if a pattern emerges for specific patient groups.

## Methods

### Dataset

The dataset used is the publicly available eICU Collaborative Research Database [10] that consist of over 200 000 patient stays from multiple intensive care units (ICU) in the US during 2014 and 2015. The dataset contains information related to admission, and intermittent and continuous information about lab values and vital values during the stay. The database includes tables dedicated to values for calculating the Acute Physiology and Chronic Health Evaluation (APACHE) IV severity-of-disease classification system [11] that also translates into a hospital mortality prediction.

### Patient selection

**Fig. 1:**
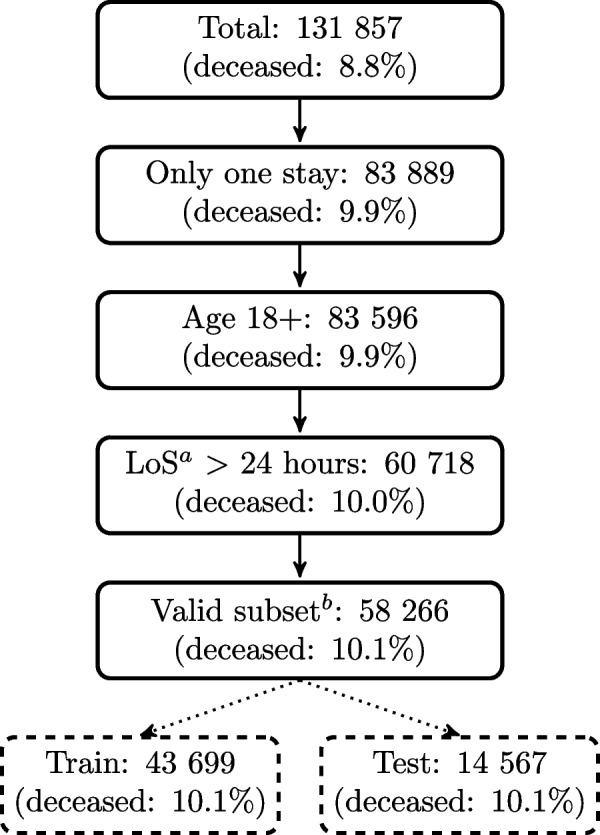
Patient selection. Patient selection criteria and train/test splitting of dataset. ^a^Length of stay. ^b^Patients with missing or unknown *age*, sex, patient id, hospital discharge status, *admissionheight*, *admissionweight*, and predicted hospital mortality. Outliers for *admissionheight* and *admissionweight* are removed

The selection of patients is shown in Fig. 1. Patients with more than one ICU stay are excluded from the study to avoid effects from treatment before or after the ICU stay in question. Patients younger than 18 years, and patients with a ICU stay shorter than 24 hours are also excluded. This last exclusion serves two purposes; ensure that the APACHE values are correct and remove patients that are in the ICU for only a short amount of time before being transferred or discharged. Patients with missing categorical variables (admission diagnosis (*adx*), or *unit admit source*) are excluded as well as patients without missing or unknown *age*, *sex*, patient id, hospital discharge status, and predicted hospital mortality. Patients with an *admissionheight* outside the range <100 cm, 250 cm> or an *admissionweight* outside <30 kg, 300 kg> are imputed with the mean of the sex as these extreme values are assumed to be artefacts. Some key patient characteristics can be found in Appendix A.

### Feature selection

The features selected are mostly the same variables as found in the APACHE tables in the eICU dataset, and comprise the physiological variables given by the APACHE tables, whether the patient has one of the chronic health conditions used for calculating APACHE IV, the unit admit source, hospital length of stay before the ICU admission, if the patient had an emergency surgery, admission diagnosis system and whether it is operative or non-operative, and the age, sex, height, and weight of the patient.

The values for the physiological variables used to calculate the APACHE IV score are the ‘worst’ value in the first 24 hours for each feature. The ‘worst’ value for each feature is defined as the value furthest away from a reference value.

### Preprocessing

The majority of ML models do not allow missing values in the training or test set. Missing numerical values are imputed with the mean of the training set. Imputation of values leads to changes in information compared with the original dataset. Which imputation method to use depends on the use case, algorithm, and the type of data available. Imputation using means is used partly for practical reasons. In addition have all continuous features with missing values a unimodal distribution, apart from *FiO2*, which makes the mean a suitable imputation option. The *FiO2* includes many missing values (73.2%), but without any clear pattern in which values are missing, the mean is considered an acceptable alternative for this study. Categorical features are one hot encoded.

### Machine learning models

Three structurally different ML algorithms are chosen to reduce model specific uncertainties: Logistic regression (LR), eXtreme Gradient Boosting (XGB), and Adaptive Boost (ADA) [12] classifier. The models are chosen as they are readily available, can achieve similar performance and are not computationally expensive. All algorithms have previously been used in mortality prediction studies [13-15]. Logistic regression resembles linear regression in calculation, but the output is restricted between 0 and 1. A restricted output makes the LR suitable as a binary classifier. The tree ensemble models, XGB and ADA, comprise multiple decision trees that determine the output by imposing a series of conditions on the input in a flowchart-like structure. The XGB algorithm minimises loss when adding new models by using a gradient decent algorithm. The ADA model decides on the output by a weighted majority vote after sequential training of multiple decision trees based on the previous tree’s errors.

### Process

**Fig. 2:**
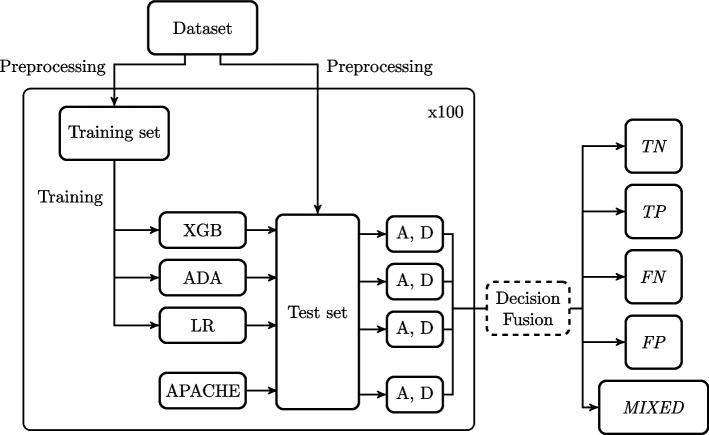
Workflow. The dataset is split into training and test sets before the ML models are developed using the training set. All models provide an ‘Alive’ (A)/‘Dead’ (D) prediction for all patients in the test set. The procedure of splitting the dataset, training the ML models and making a prediction of all the patients in the test set is then repeated 100 times. All results for each patient are combined and the patients are placed in one of the final groups (*TN*, *TP*, *FN*, *FP*, or *MIXED*) depending on the results. This decision fusion is shown in Fig. 3

**Fig. 3:**
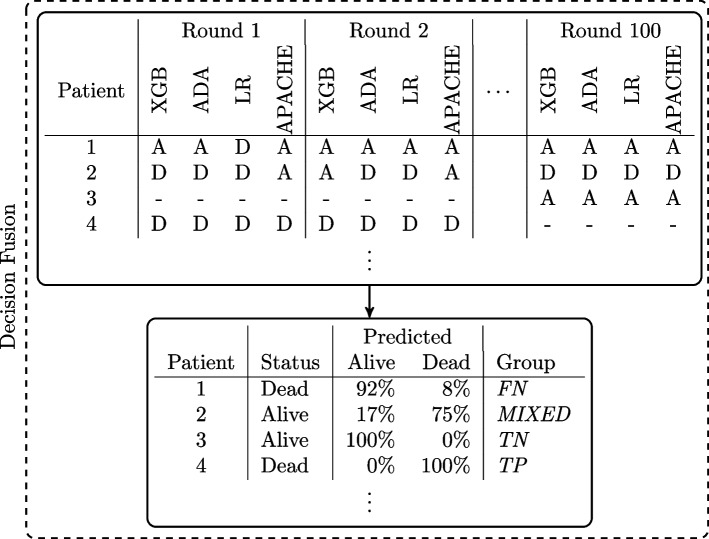
Decision Fusion. Results from all rounds and models are combined, as shown in the upper table. The table shows the results from four fictive patients. The A and D represent ‘Alive’ and ‘Dead’, respectively. The hyphens ‘-’ represent the rounds where the patient is not in the test set. Patient 1 is predicted alive at discharge by three models for round 1, while the LR model predicts dead at discharge. All models predict alive at discharge for round 2 and round 100. Round 3-99 are not shown. The lower table shows the patient’s discharge status, the percentage of predicted ‘Alive’ and ‘Dead’ across all models and rounds, and the final group. The percentage of ‘Alive’ predictions is 92%, and ‘Dead’ is 8%. With an agreement threshold of 90%, the models agree on an ‘Alive’ prediction. However, as seen in the lower table, the patient is dead at discharge, thus, is placed in the *FN* group. Patient 2 is placed in the *MIXED* group since neither ‘Alive’ nor ‘Dead’ are predicted more than 90% of the patient’s occurrences in the test set. Patients 3 and 4 are placed in the *TN* and *TP* group respectively since all models agree on the prediction across all rounds, and the prediction is the same as the discharge status

The workflow is shown in Figs. 2 and 3. After the patient selection shown in Fig. 1, the dataset is divided into a train and a test set with a similar share of alive and dead patients (10.5%). The split is 75% of the patients for the training set and 25% for the test set. Hospital discharge status is the end outcome, and the models are trained to predict whether a patient is alive or dead at hospital discharge.

The models are trained on the training set using grid-search for hyperparameter optimisation (with area under the receiver operating characteristics curve as scoring), and predictions between 0 (alive) and 1 (dead) are made for the patients in the test set. The continuous predictions of the patients in the test set are translated into an ‘Alive’ or ‘Dead’ prediction based on a threshold given by the maximum Youden’s index (*J*) [16] for each model. The Youden’s index is given by the formula:

$$\begin{aligned} \quad \quad \quad \quad \quad \quad \quad \textrm{J} = \text {sensitivity + specificity - 1.} \end{aligned}$$

The procedure of splitting the data into train and test sets, training the ML models, and predicting the outcome of the patients in the test set is repeated 100 times which results in 100 slightly different models per algorithm, with corresponding results. The repetition of this procedure allows us to investigate whether a patient’s predicted outcome is the same regardless of the model used or if the prediction depends on the applied ML model. Each of the 3x100 ML models can be considered an independent model and none of the models are influenced by the training or testing of any of the other repetitions.

The number of predictions per patient depends on the number of times the patient is placed in the test set. For each time in the test set, the patient gets four new predictions: one from each of the three ML models and one from the APACHE model. The APACHE prediction is constant for each patient in the dataset and does not change with the train/test split. After the 100 rounds, the predictions from all rounds and models are combined and a final prediction is decided with an agreement threshold. If X% of the models predict ‘Alive’, and X% is above the agreement threshold, the final prediction is ‘Alive’. If Y% of the models predict ‘Dead’, and Y% is above the agreement threshold, the final prediction is ‘Dead’. If neither X% nor Y% is above the agreement threshold, the result is ambiguous and the patient is marked with a *MIXED* prediction.

After receiving a final prediction, the patients are divided into groups based on the final prediction and hospital discharge status as shown in Fig. 3. If the final prediction is that the patient is discharged alive, and the patient is discharged alive, the patient is placed in the ‘true negative’ (*TN*) group. If a patient dies in the hospital, but the final prediction is that the patient is discharged alive, the patient is placed in the ‘false negative’ (*FN*) group. The other groups are ‘true positive’ (*TP*), and ‘false positive’ (*FP*). The patients with a *MIXED* final prediction is placed in the *MIXED* group.

The area under the receiver operating characteristics (AUROC) curve and area under the precision recall curve (AUPRC) are commonly used performance metrics on dataset-level and are used to evaluate the performance of each model in terms of how well the models are able to discriminate between the two outcomes. The AUROC is the false positive rate plotted against the true positive rate for different model decision thresholds, and the value varies between 0.5 (a random classifier) and 1 (a perfect classifier). The baseline of the AUPRC depends on the share of positive samples in the dataset. The AUPRC is more descriptive for unbalanced datasets but is less reported in studies. The Brier score is a score for evaluating a models calibration in combination with discrimination, i.e. how well the model predictions match real world probabilities. The Brier score can be calculated by taking the mean square error of the prediction and the result is given as a score between 0 and 1. Lower numbers are preferred.

### Feature importance

Feature importance helps estimating how much each feature contributes to the prediction. The feature importances are found using SHapley Additive exPlanations (SHAP) [17] which is a method for explaining individual predictions based on Shapley values [18]. Shapley values give the average marginal contributions for each participant in a game, which in this case is the contribution of each feature on the final prediction. The feature importances are found for each ML model before the contributions are compared. The average feature importance is calculated by normalising feature importance for each prediction. The contribution of each feature is then averaged for each patient before the average of all patients is calculated. The results for the categorical variables *adx* and *unit admit source* are found by combining the feature importance from the individual categories.

### Comparison of predictions

The groups *TN*, *TP*, *FN*, *FP*, and *MIXED* are compared to find differences between consistently correctly and incorrectly classified patients. The feature importances provide a basis for the focus of the rest of the analysis. Continuous numerical variables are compared using histograms to see whether the distribution of feature values differs between the groups. Kernel density estimation (KDE) is used to create density curves by replacing each sample with a kernel before summing over all replacements. The density curves are used for simpler comparison between groups. Binary and categorical variables are compared using relative occurrences.

### Software

The study uses Python 3 [19] programming language for development and analysis of models and results. The ADA and LR algorithms are available with the Scikit learn [20] library and the XGB algorithm is available with the XGboost package with scikit-learn interface [21]. SHAP values are calculated using the SHAP library [17]. Figures are created with the Matplotlib library [22].

## Results

### General information

During the 100 rounds, each patient occurred in the test set minimum 8 times, and maximum 46 times. The mean number of occurrences was 25.

**Fig. 4:**
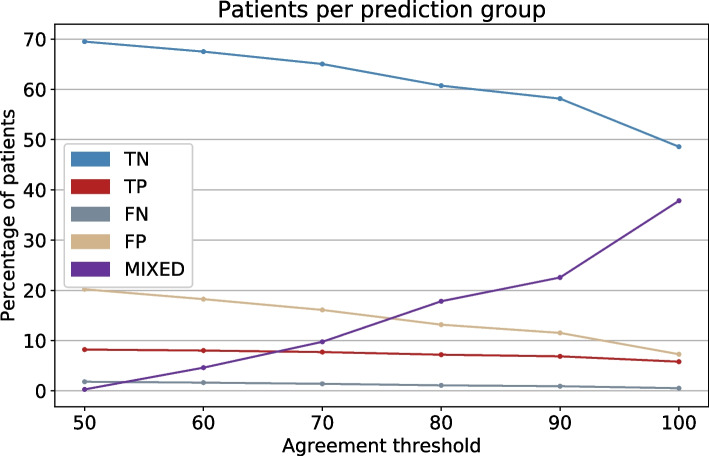
Patients per group. The percentage of patients in the groups *TN*, *TP*, *FN*, *FP*, and *MIXED*. The percentage of agreement is given by the x-axis

Figure 4 shows the number of patients per group for different agreement thresholds. The y-axis represents the percentage of patients, and the x-axis shows the threshold that decides the final prediction. The *TN* group is the largest group for all combinations. The *MIXED* group has the biggest change in the number of patients, depending on the threshold.

The selected threshold for further analysis is set to 90%. This threshold is selected as a trade-off between confident groups and a sufficient number of patients in each group for analysis.

The number of patients per category is listed in Table 1. Out of the 13 108 patients in the *MIXED* category, 11 779 (89.9%) were alive at hospital discharge, and 1 329 (10.1%) dead.

**Table 1:** Number of patients per group

*TN*	34 056 (58.4%)
*MIXED*	13 108 (22.5%)
*FP*	6 572 (11.3%)
*TP*	3 984 (6.8%)
*FN*	546 (0.9%)

### Performance metrics

The AUROC, AUPRC, and Brier score are calculated for each model for each of the 100 rounds. The mean and standard deviation are listed in Table 2.

**Table 2:** Average model performance

Model	Mean AUROC [std]	Mean AUPRC [std]	Mean Brier score [std]
XGB	0.872 [0.004]	0.488 [0.012]	0.068 [0.001]
ADA	0.870 [0.003]	0.475 [0.012]	0.238 [0.004]
LR	0.858 [0.004]	0.434 [0.013]	0.153 [0.002]
APACHE IV	0.862 [0.004]	0.465 [0.011]	0.070 [0.001]

Considering each round individually, the XGB and ADA models have a higher AUROC than the LR and APACHE models for all rounds. The XGB models have a higher AUROC than the ADA models for most rounds, and the APACHE model has a higher AUROC than the LR models for the majority of the rounds. Considering the AUPRC for each round individually, the LR models perform significantly worse than the other models. The XGB models have the overall best performance in terms of discrimination, followed by the ADA and APACHE models. The calibration of the XGB and APACHE models are quite similar, and better than the calibration of the LR and ADA models. A subgroup analysis for *sex* and *age* can be found in Appendix B.

### Feature importance

Table 3 shows the 15 most important features for each algorithm, listed with decreasing importance. The feature importances are listed using the average normalised feature importance, given in the parenthesis behind each feature.

**Table 3:** Feature importance rank for the machine learning models. The mean contribution for each feature as percentages is shown in parentheses. The combined contribution of the top 5, 10, and 15 feature are shown in bold face

Rank	XGB	ADA	LR
1	Age (0.096)	Age (0.106)	Age (0.096)
2	Vent (0.089)	Vent (0.077)	Source$^b$ (0.084)
3	Verbal (0.077)	HR (0.068)	Vent (0.075)
4	BUN (0.068)	BUN (0.057)	Adx (0.067)
5	Motor (0.058)	Elective$^a$ (0.051)	HR (0.061)
**Top 5**	**0.390**	**0.360**	**0.382**
6	HR (0.055)	Temp (0.051)	Verbal (0.057)
7	RR (0.049)	Verbal (0.050)	RR (0.053)
8	WBC (0.041)	WBC (0.049)	Eyes (0.051)
9	Temp (0.040)	RR (0.049)	BUN (0.045)
10	Adx (0.038)	Source$^b$ (0.043)	Op/Non-Op (0.040)
**Top 10**	**0.613**	**0.602**	**0.628**
11	Elective$^a$ (0.034)	Motor (0.036)	Motor (0.039)
12	Mean BP (0.028)	Adx (0.034)	Albumin (0.038)
13	Source$^b$ (0.028)	FiO2 (0.29)	WBC (0.030)
14	Creatinine (0.026)	Mean BP (0.029)	Intubated (0.029)
15	Eyes (0.026)	Sodium (0.028)	Temp (0.027)
**Top 15**	**0.754**	**0.758**	**0.791**

*Abbreviations* *Adx *Admission diagnosis, *BP *Blood pressure, *HR *Heartrate, *Op/Non-Op *Operative/Non-operative, *RR *Respiratory rate, *Temp *Temperature

$^a$Elective surgery, $^b$Unit admit source

The algorithms agree on two of the top five features, six of the top ten features and eleven of the top 15 features. The five most important features contribute to 38% of the predictions on average, while the top 15 features for each model contribute to 77% on average.

**Fig. 5:**
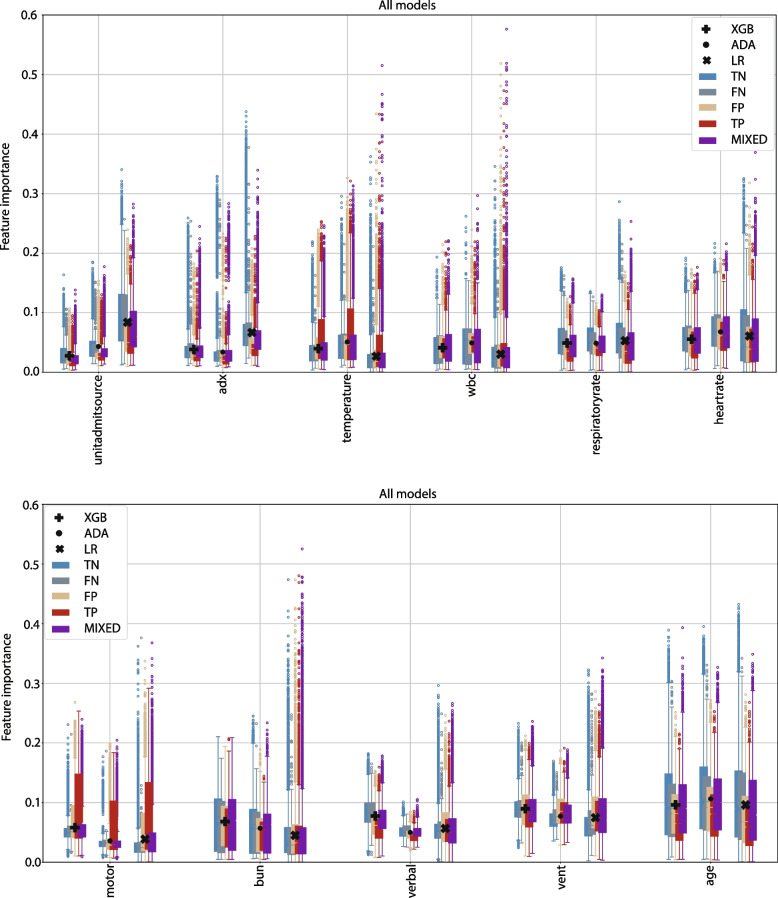
Feature importance. Each point represent the average feature importance for each iteration, feature, ML algorithm, and group. The vertical clusters of groups marked with either a plus, dot or x, represent the different ML algorithms

The feature importances are found for all patients in the test sets, as well as alive and dead patients, and the groups *TN*, *TP*, *FN*, and *FP* separately to determine potential differences between them. Figure 5 shows the feature importances for each model for the common numerical features among the top 15 features. The colours represent the different groups. Each data point represents the average feature importance for each round for each algorithm and group. The black markers represent the mean feature importance across all rounds and groups. The three clusters of box plots for each feature represent the three different ML algorithms. The XGB being the leftmost cluster, ADA in the middle, and the LR model the rightmost one, all represented by different marker types.

### Most important common features

**Fig. 6:**
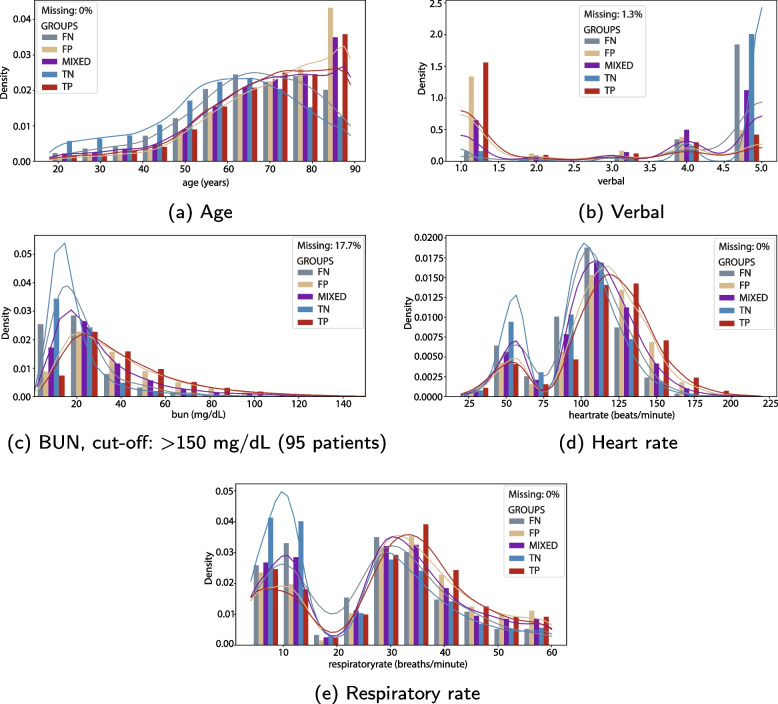
Histogram and kernel density plots. The histogram and kernel density plots for the five top common numerical (not binary) features for the different groups (*TN*, *TP*, *FN*, *FP*, *MIXED*). The feature value is shown along the x-axis. For features with large outliers are the x-axes limited by a cut-off value. The number of patients not shown in the figure is given in the parentheses

Histograms and kernel density estimate (KDE) curves showing the densities for the top five common variables *age*, *verbal*, *bun*, *heartrate*, and *respiratoryrate* are shown in Fig. 6. The histograms for features considered less important are shown in Appendix C.

Whether the patient was ventilated (*vent*) at the time of the worst respiratory rate is also considered an important factor. The percentages for each group for the *vent* feature is given in Table 4. The percentage of *TP* patients that were ventilated is 69.1%, which is the highest percentage of the groups. The second highest value is 60.5% for the *FP* patients, followed by *MIXED*, *FN* and *TN*.

**Table 4:** Share of patients ventilated at the time of the worst respiratory rate for each group

*vent*	FN	FP	MIXED	TN	TP
**No**	83.5 %	39.9 %	64.4 %	88.2 %	30.7 %
**Yes**	16.5 %	61.1 %	35.6 %	11.8 %	69.3 %

Many patients have one or more missing feature values. While all patients have a feature value for *heartrate* and *respiratoryrate*, less than 73% of the patients have values for *pH* and *FiO2*. The histograms show the values present in the dataset, and imputed values are not included. Thus, the histograms show the actual patient values but do not contain all information used in the training and testing of the models.

### Additional parameters

**Fig. 7:**
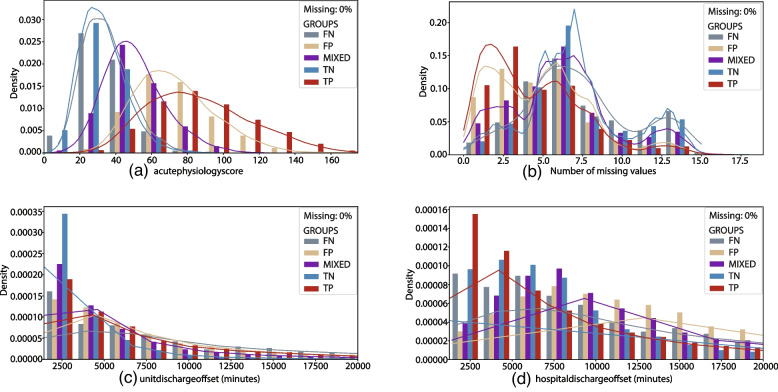
Histogram and kernel density plots. Features not used directly as input to the model. The histogram and kernel density plots for the different groups (*TN*, *TP*, *FN*, *FP*, *MIXED*). The feature value is shown along the x-axis. For features with large outliers are the x-axes limited by a cut-off value. The number of patients not shown in the figure is given in the parentheses

Figure 7 visualises the unit discharge offset, hospital discharge offset, acute physiology score (APS), and the number of missing feature values. These parameters are not included directly as features in the model, but are relevant for the comparison of the different groups.

The time from unit admission to unit discharge is shown in Fig. 7c, and the time from unit admission to hospital discharge is shown in Fig. 7d. The histograms only show stays up to 20 000 minutes (about two weeks). The unit discharge offset and the hospital discharge offset are identical for 12%-14% of the patients in the *TN*, *FP* and *MIXED* groups. For the *TP* and *FN* groups are the percentages of patients with identical unit and hospital discharge offset 44% and 38%, respectively.

## Discussion

### General information

Considering the patients the models agreed on, the number of patients with prediction ‘Dead’ is 23.4%, which is considerably larger than the mortality in the complete data set. The *FN* group is minuscule compared with the total dataset, and the generalisability of this group is debatable. The share of dead patients in the *MIXED* group is slightly higher than in the whole dataset as seen in Table 1.

### Performance metrics

The XGB models performs better in terms of AUROC, AUPRC, and Brier score, followed by the ADA models (apart from the Brier score) the APACHE IV, and the LR models. The models have similar population-level performance in terms of AUROC, and are comparable to previous literature [23]. The APACHE IV AUROC is lower compared with the original study with an AUROC of 0.88 [11].

### Feature importance

The feature importance values listed in Table 3 show how much each feature contributes to a prediction on average, and the importance of individual features can vary significantly between patients. The algorithms agree to a large extent on which features are most important for the mortality prediction, which suggests that these features are important concerning mortality in a clinical setting, and not for the ML models alone.

Binary variables are generally considered less important by the models, with the exception of whether the patient was ventilated at the time of the worst respiratory rate. Most of these variables could be relevant for mortality of individual patients but are not considered important for the overall patient population. This could be because the models do not consider the variables important, or because the number of patients where the variable is important is small compared with the population.

The interquartile ranges of the different prediction groups are overlapping for all features (Fig. 5), and there are no clear differences between the prediction groups or algorithms. The large variance and many outliers indicate large individual differences.

Despite knowledge about which features impact the prediction, we cannot use the information from this study to determine the feature values. A high feature value may correlate to high feature importance, and a low feature value to low feature importance, or vice versa. Such relationships cannot be understood from Fig. 5 or Table 3.

### Most important common features

The shape of the KDE curves depends heavily on the type of variable. Many of the curves follow the shape expected for the specific type of feature, but since the values used are the most abnormal ones in the first 24 hours, vital values as heart rate and mean blood pressure (Fig. 6) have less intuitive distributions with two distinct peaks. Vital values may differ much during 24 hours, regardless of health status, and few patients have exclusively measurements proximate to the reference value.

Considering the plots in Fig. 6, the *TN* KDE curves are higher and narrower than the other groups for all features apart from *age*. The *TN* group contains more patients, and many of these patients have values close to the reference value used to calculate the ‘worst’ values. The *TP* and *FP* curves are more similar to each other than to the other groups, which indicates that patients in those two groups have more similar characteristics.

The distributions for the rest of the numerical, non-binary features in Appendix C also differ. No simple pattern can represent all of them. In general, the curves for the *TP* and *FP* groups are flatter than the *TN* and *FN* curves, which could be explained by a larger share of patients with more extreme values in the *TP* and *FP* groups. Many of the continuous variables deemed less important for the prediction have either many missing values (e.g., *albumin* and *pH*), and/or similar KDE curves (e.g. *admissionheight*).

The binary variable *vent* support the idea that patients with the same predicted outcome have more similar feature values. The results also suggest that *TP* patients are sickest, followed by *FP*, *MIXED*, *FN* and *TN*.

### Additional parameters

The APS is a weighted score of several physiological values and is a component of the APACHE score. The score is calculated based on the most abnormal values in the first 24 hours. The higher the value, the sicker the patient. Figure 7a, displaying the APS density histograms, shows that patients in the *TN* and *FN* groups generally are less ill than patients in the other groups, followed by *MIXED*, *FP*, and *TP*. This order is intuitive; less sick patients are classified as alive at discharge, while sicker patients are classified as dying in the hospital. The patients in the *MIXED* group are somewhere in between.

Missing values are imputed with the mean of the training set. Figure 7b shows the number of missing values per patient for the different groups. Values are most likely not missing completely at random, and the missing values may contain information themselves. Patients classified as dying have a lower number of missing values than the other patients that may result from sicker patients requiring more testing the first 24 hours. However, the missing values does not necessarily affect the predictions, and the effect of this is not investigated in this study.

The patients classified correctly have a shorter hospital stay than the patients classified incorrectly. The vital and lab values used for predictions stem from the first 24 hours of the ICU stay and become less relevant for more prolonged hospital stays. Patients in the *TN* group are discharged from the ICU earlier than the other groups, while *TP*, *FP* and *FN* are likely to have a longer length of stay. The distributions of hospital length of stay differ from the distributions of unit length of stay. Considering both graphs, we can see that patients classified as *TN* have a short unit length of stay, but are discharged later from the hospital. A large share of the patients that die in the hospital die in the ICU.

### Clinical relevance and implications

In a clinical setting, the correct classification of patients is crucial. The number of patients in each group for a given model is dependent on both the threshold value for the *MIXED* group and a trade-off between sensitivity and specificity from the respective ROC-curves. The overall performance of our analysis, at least from the tree-based models, are slightly better than the APACHE IV score.

As seen from Fig. 4, the true negative group is large with a high specificity with a small false negative group. This is true even at a low agreement threshold between iterations at 50%. This means that the algorithm in clinical practice will have a very high negative predictive value: If the algorithm states that the patient will be discharged alive, he/she probably will. This is more problematic with the positive groups with the false positive being twice as large as the true positive. This means that the positive predictive value is low: If the algorithm states that the patient will die, the likelihood for this being true is only approximately 30%. Interestingly, by increasing the agreement threshold from 50 to 100%, the false positive group more than halves while the true positive group only slightly decreases. For practical purposes, this means that if the patient is classified as negative one should trust this with a low agreement threshold (increasing the threshold will have many patients moving from the negative to mixed group). On the other hand, if the patient is classified as positive, the agreement threshold should be increased to minimise the size of the false positive group and thus increase the positive predictive value.

From all the measured variables, all three algorithms agree upon the patient *age* being the most important predicting feature. Being connected to a ventilator is the second most important feature in two of the models, and the third most important in the two others. The models agree less on the importance of other features. BUN values (indicating renal failure) are important in some models and values derived from the Glasgow Coma Scale (*eyes*, *verbal*, *motor*) are in others. One could think that complicated lab tests and information like admission diagnosis and unit admit source would be important features and add a lot to the models, but it does not. Likewise, for one of the features that really concerns ICU staff, the *FiO2*, which is only in the top 15 list in one of the models.

One explanation for the lack of importance of features generally considered as important by clinicians could be the number of missing values in the dataset, e.g., 73% of patients do not have a *FiO2* value. The issue of missing values is to be considered a general problem of ‘clinical’ dataset [24], and using a more complete dataset could possibly give explanations closer to one of a medical understanding.

We state in the background that there is a lack of trust in ML models in clinical medicine, and our findings somewhat strengthen that distrust. As many as 22% of our models were inconsistent classified with different seeds iterations, and many of those who were consistent classified were wrong, 11.5% false positive and 0.9% false negative. It is difficult to know how much of the problem is caused by aleatoric vs epistemic uncertainty, but the first step to move forward is more comprehensive dataset; we will never know whether a “complete” eICU set would have gained significant better results. In order for clinicians to trust the models they first need to perform better, and secondly to be more explainable as earlier explored by our group [25].

### Limitations and future work

Missing values is a common problem when working with ML using real life datasets. The impact of the missing values on the results differ depending on how they are dealt with and the use of the ML model. This study imputes missing values with the mean of the training set. However, the results would most likely differ for both feature importance and density curves if the same study is conducted on a dataset with no missing values. When the number of missing values is high and the missing values are imputed with mean, the patients appear more similar to each other. A possible consequence of this is that the feature is deemed less relevant since it is harder to separate the patients using the specific feature. A separate study should be conducted to investigate the importance of the missing values to gain better understanding.

This study investigates the importance of individual features alone. Future studies should also include the importance of combinations of features, as well as the feature values. In addition to a greater understanding of the models and how and why they make certain predictions, this can help with identifying incomplete coverage of the domain, which in turn may reduce the epistemic uncertainty for future models. In addition, an analysis by medical personnel could be conducted to see they find the same patients hard to classify.

Classification thresholds are used to divide patients into TP, TN, FP, and FN groups. How certain a model is about the prediction is not taken into account and a patient with a prediction of 0.01 and a patient with a prediction of 0.49 would get the same ‘Alive’ prediction with a classification threshold of 0.5. Doing similar analyses with well-calibrated models and without using classification thresholds is an interesting focus for future studies.

Another limitation is that the results are found using only one database. While the database includes information about many different patients from multiple hospitals, factors concerning patient population and treatment may differ from other ICUs. The results should not be considered universal for all patient populations.

## Conclusion

This study shows that differences between patients correctly and incorrectly classified are small, or even overlap, and that individual features cannot be used to separate these patient groups. Using the weighted APS score, differences between the groups are clearer, which indicate that a combination of features could be a focus for future studies. The features of incorrectly classified patients are more similar to the features of patients with the same prediction rather than the same outcome; *FN* patients are generally healthier than *TP* patients, and *FP* patients are sicker than *TN* patients. The *MIXED* group is in between. While adding more features to better distinguish these patients could improve the results, it could be that the features are too similar to distinguish, or that the uncertainties related to the outcome are too large despite increased knowledge.

How to utilise this information to further improve models in health care depends on the use case. While patients in the *MIXED* group can be flagged with an uncertain prediction, the same cannot be done with patients with a false prediction. Almost all patients predicted to be alive at discharge are alive at discharge, while more than half of the patients predicted to die are discharged from the hospital alive.

A focus on which samples a model work/does not work for should be a concern for all use cases of ML models. This paper highlights some important issues associated with the development of ML models in health care settings, and illustrates limitations frequently neglected in ML model performance analyses. Discerning which samples a model is reliable for is useful with further development of the model, and essential before deploying it.

## Supplementary Information

**Additional file 1.**

## Data Availability

The dataset used is from the publicly available eICU Collaborative Research Database v2.0 [10]. The code for reproducing the work in this study will be published on GitHub (https://github.com/Explainable-Hospital-Mortality/comparison-correctly-incorrectly-predictions).

## Abbreviations

ADA: Adaptive Boosting,
APACHE: Acute Physiology and Chronic Health Evaluation,
APS: Acute Physiology Score,
AUPRC: area under the precision recall curve,
AUROC: Area under the receiver operating characteristics curve,
BUN: Blood Urea Nitrogen,
FN: False Negative,
FP: False Positive,
ICU: Intensive Care Unit,
KDE: Kernel Density Estimate,
LR: Logistic Regression,
ML: Machine Learning,
SHAP: SHapley Additive exPlanations,
TN: True Negative,
TP: True Positive,
XGB: eXtreme Gradient Boosting
