# Supplementary material for: Comparison of correctly and incorrectly classified patients for in-hospital mortality prediction in the intensive care unit
Source: BMC Med Res Methodol. 2023 Apr 24;23:102. doi: 10.1186/s12874-023-01921-9 (PMC10124049; doi:10.1186/s12874-023-01921-9)
Supplement: Supplementary file 1 — Additional file 1. [file 12874_2023_1921_MOESM1_ESM.zip › Appendices.pdf]

Appendix A: Key Patient Characteristics

Table 1: Key patient characteristics.

	Overall (n = 58 266)	Alive (n = 52 407)	Dead (n = 5 859)
**Age (years), median [IQR]**	65 [53-77]	65 [53-76]	71 [60-81]
<50 years, n (%)	11091 (19.0%)	10479 (20.0%)	612 (10.4%)
50-80 years, n (%)	35705 (61.3%)	32151 (61.3%)	3554 (60.7%)
≥80 years, n (%)	11470 (19.7%)	9777 (18.7%)	1693 (28.9%)
**Male, n (%)**	31 799 (55%)	28 628 (55%)	3 171 (54%)
**Admission diagnosis, n (%)**			
Cardiovascular	26 066 (44.7%)	22 986 (43.9%)	3 080 (52.6%)
Neurologic	10 912 (18.7%)	10 051 (19.2%)	861 (14.7%)
Respiratory	7 698 (13.2%)	6 672 (12.7%)	1 026 (17.5%)
Gastrointestinal	5 868 (10.1%)	5 410 (10.3%)	458 (7.8%)
Trauma	3 117 (5.3%)	2 872 (5.5%)	245 (4.2%)
Metabolic/Endocrine	2 027 (3.5%)	1 974 (3.8%)	53 (0.9%)
Genitourinary	1 460 (2.5%)	1 375 (2.6%)	85 (1.5%)
Musculoskeletal/Skin	666 (1.1%)	643 (1.2%)	23 (0.4%)
Hematology	368 (0.6%)	340 (0.6%)	28 (0.5%)
Transplant	84 (0.1%)	84 (0.2%)	0 (0%)
**Length of stay (days)**			
ICU, median [IQR]	2.2 [1.6-3.9]	2.1 [1.5-3.7]	3.6 [2.0-6.8]
Hospital, median [IQR][a]	5.8 [3.4-9.6]	5.9 [3.5-9.5]	5.3 [2.8-10.1]

[a]615 patients are missing the *hospitaladmitoffset* value.

Appendix B: Subgroup analysis

A subgroup analysis is done for *sex* and *age*. The results can be found in Tables 2 and 3 for *sex* and Tables 4 and 5 for *age*. For *sex*, the share of patients in the FN, FP, MIXED, TN, and TP groups are similar for males and females as seen in Table 2. There are minor differences in performance metrics, as seen in Table 3, which can be explained by small differences in the datasets. For *age*, the differences between the groups are larger. The share of patients in the FN, FP, MIXED, TN, and TP groups are similar for the three age groups for FN, while the share differs for the other groups as seen in Table 4. There are also differences between the groups in terms of performance metrics, as seen in Table 5. The age group $<$50 years has significantly better AUROC scores than the other age groups and the group $\geq$80 years performs worse than the overall population. One explanation for this is the difference in the share of alive and dead patients for the different age groups as seen in the table in Appendix A, but this should be investigated further in future studies.

Table 2: The share of patients in the FN, FP, MIXED, TN, and TP groups per sex. The rows summarise to 100%.

Sex	**FN**	**FP**	**MIXED**	**TN**	**TP**
**Male**	0.9 %	10.8 %	21.7 %	59.8 %	6.8 %
**Female**	0.9 %	11.8 %	23.5 %	56.9 %	6.9 %

Table 3: Average model performance for sex subgroups.

Sex	Model	Mean AUROC [std]	Mean AUPRC [std]	Mean Brier score [std]
Female	XGB	0.867 [0.006]	0.484 [0.018]	0.069 [0.002]
	ADA	0.866 [0.006]	0.465 [0.017]	0.239 [0.004]
	LR	0.856 [0.007]	0.427 [0.019]	0.156 [0.003]
	APACHE IV	0.854 [0.006]	0.455 [0.016]	0.073 [0.002]
Male	XGB	0.876 [0.005]	0.493 [0.017]	0.067 [0.002]
	ADA	0.873 [0.005]	0.483 [0.016]	0.238 [0.004]
	LR	0.859 [0.006]	0.441 [0.016]	0.151 [0.002]
	APACHE IV	0.868 [0.006]	0.474 [0.017]	0.069 [0.002]

Table 4: The share of patients in the FN, FP, MIXED, TN, and TP groups per age group. The rows summarise to 100%.

Age	**FN**	**FP**	**MIXED**	**TN**	**TP**
**$<$50 years**	0.8 %	4.4 %	13.6 %	77.9 %	3.3 %
**50-80 years**	1.0 %	10.4 %	21.6 %	60.2 %	6.7 %
**$\geq$80 years**	0.9 %	20.5 %	33.8 %	34.3 %	10.5 %

Table 5: Average model performance for age subgroups.

Age	Model	Mean AUROC [std]	Mean AUPRC [std]	Mean Brier score [std]
$<$50 years	XGB	0.911 [0.010]	0.499 [0.034]	0.037 [0.002]
	ADA	0.908 [0.009]	0.447 [0.035]	0.235 [0.005]
	LR	0.895 [0.010]	0.399 [0.039]	0.082 [0.003]
	APACHE IV	0.907 [0.010]	0.477 [0.036]	0.038 [0.002]
50-80 years	XGB	0.875 [0.005]	0.500 [0.015]	0.066 [0.001]
	ADA	0.873 [0.004]	0.487 [0.015]	0.238 [0.004]
	LR	0.861 [0.005]	0.448 [0.015]	0.149 [0.002]
	APACHE IV	0.865 [0.005]	0.484 [0.015]	0.068 [0.001]
$\geq$80 years	XGB	0.805 [0.009]	0.457 [0.022]	0.103 [0.003]
	ADA	0.807 [0.009]	0.462 [0.021]	0.242 [0.003]
	LR	0.791 [0.010]	0.423 [0.023]	0.235 [0.005]
	APACHE IV	0.787 [0.010]	0.423 [0.020]	0.110 [0.003]

Appendix C: Histogram and kernel density plots

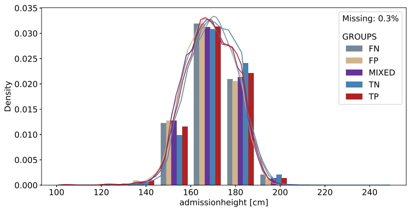

(a) Admission height

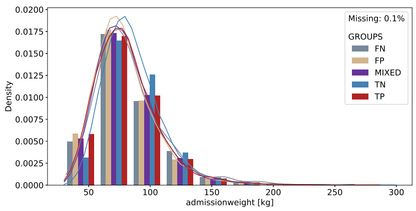

(b) Admission weight

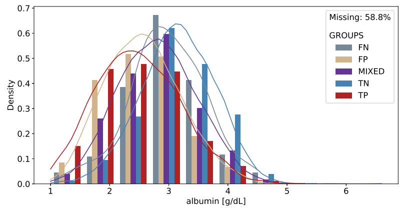

(c) Albumin

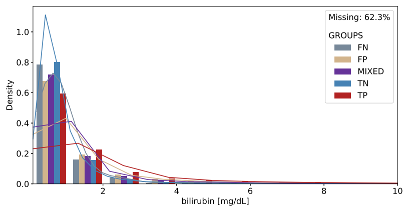

(d) Bilirubin, cut-off: >10 mg/dL (262 patients)

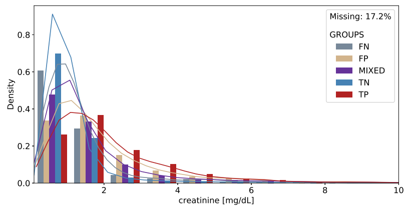

(e) Creatinine, cut-off: >10 mg/dL (368 patients)

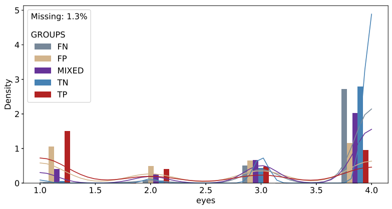

(f) Eyes

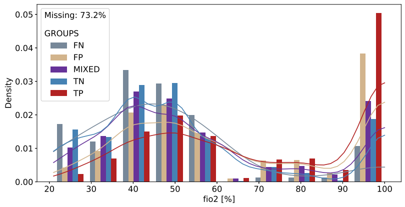

(g) FiO2

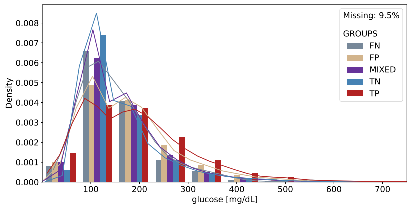

(h) Glucose, cut-off: >750 mg/dL (142 patients)

Figure 1: **Histogram and kernel density plots.** The histogram and kernel density plots for less important features for the different groups (*TN*, *TP*, *FN*, *FP*, *MIXED*). The feature value is shown along the x-axis. For features with large outliers are the x-axes limited by a cut-off value. The number of patients not shown in the figure is given in the parentheses.

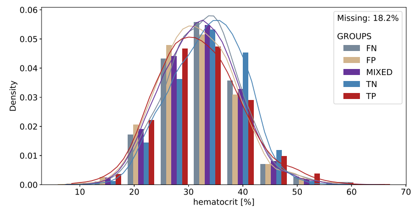

(a) Hematocrit

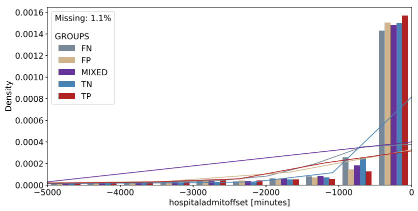

(b) Hospital admit offset, cut-off: <-5000 minutes (3935 patients)

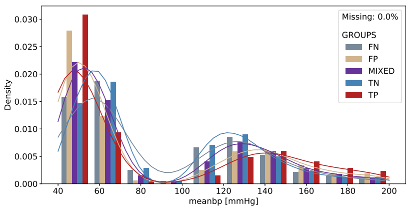

(c) Mean blood pressure

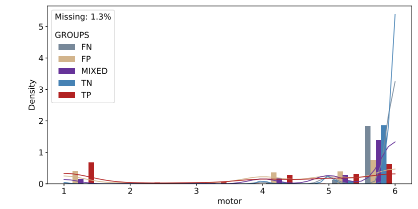

(d) Motor

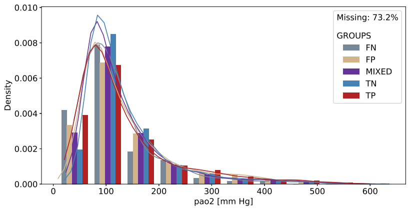

(e) PaO2

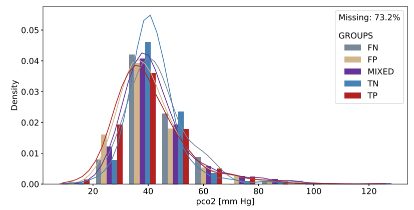

(f) pCO2

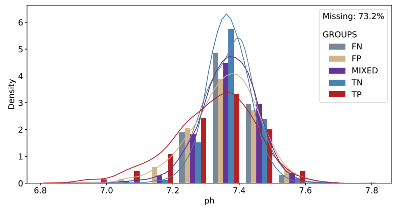

(g) pH

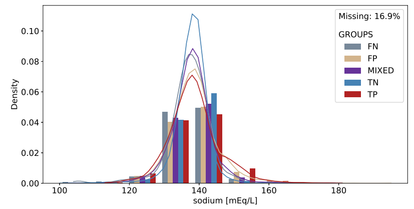

(h) Sodium

Figure 2: **Histogram and kernel density plots.** The histogram and kernel density plots for less important features for the different groups (*TN*, *TP*, *FN*, *FP*, *MIXED*). The feature value is shown along the x-axis. For features with large outliers are the x-axes limited by a cut-off value. The number of patients not shown in the figure is given in the parentheses.

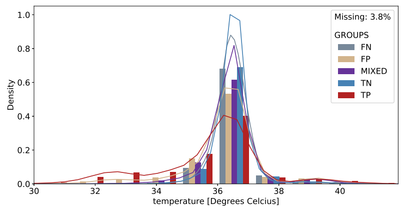

(a) Temperature, cut-off: <30 °C (83 patients)

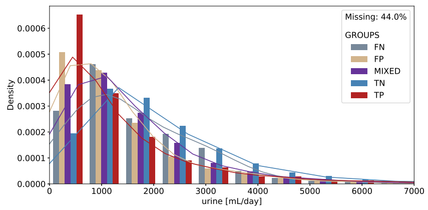

(b) Urine, cut-off: >7000 mL/day (367 patients)

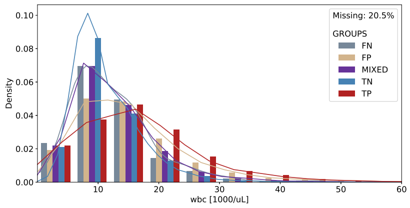

(c) White blood cells, cut-off: >60 1000/uL (107 patients)

Figure 3: **Histogram and kernel density plots.** The histogram and kernel density plots for less important features for the different groups (*TN*, *TP*, *FN*, *FP*, *MIXED*). The feature value is shown along the x-axis. For features with large outliers are the x-axes limited by a cut-off value. The number of patients not shown in the figure is given in the parentheses.
